# A Multilevel Approach to Understanding the Determinants of Maternal Harsh Parenting: the Importance of Maternal Age and Perceived Partner Support

**DOI:** 10.1007/s10826-021-01990-8

**Published:** 2021-06-08

**Authors:** Laura Farley, Bonamy R. Oliver, Alison Pike

**Affiliations:** 1 Department of Psychology, School of Arts and Social Sciences, City, University of London, Northampton Square, London, EC1V 0HB UK; 2 Department of Psychology and Human Development, UCL Institute of Education, University College London, 24-26 Woburn Square, London, WC1H 0HT UK; 3 School of Psychology, University of Sussex, Sussex House, Falmer, BN1 9QH UK

**Keywords:** ALSPAC, Parenting, Harsh discipline, Partner support, Multilevel modeling

## Abstract

Determinants of parenting are most often considered using one child per family within a cross-sectional design. In 182 families, the current study included two siblings and sought to predict maternal harsh parenting measured prospectively when each child was age 2 years from child gender, infant temperament, maternal age, maternal educational attainment, maternal depression and anxiety and maternal perceptions of partner support. Multilevel modeling was used to examine between- and within-family variance simultaneously. Mothers reported levels of harsh parenting that were similar towards both children (intraclass correlation = 0.69). Thus, the majority of variance in maternal perceptions of their harsh parenting resided between rather than within families and was accounted for in part by maternal age and maternal perceptions of partner support. Results are discussed in relation to family-wide determinants of harsh parenting, previous literature pertaining to parenting siblings and the potential avenues for future research and practice.

## Highlights

- Determinants of harsh parenting examined within a prospective, longitudinal sibling study.
- Multilevel design for simultaneous consideration of child-specific and family-wide predictors.
- Family-wide determinants were dominant, over and above child characteristics.
- Implications of perceptions of partner support discussed.
- Importance of considering parenting determinants within a sibling design emphasized.

The key role that parents play in children’s social adjustment has been understood for some time, with decades of research implicating parental sensitivity and warmth for children’s well-being as well as for promoting prosocial behaviour (Maccoby, 2015). Research has indicated that harsh parenting practices such as yelling and smacking can have substantial negative effects on child adjustment (Deater-Deckard & Dodge, 1997; Glascoe & Leew, 2010; Mammen et al., 2002). Specifically, harsh verbal and physical discipline are associated with increases in child and adolescent internalizing and externalizing symptoms, with only a minimal buffering effect of positive parenting behaviors such as warmth (McKee et al., 2007). Moreover, the early toddler years confer significant risk for the development of these impactful parenting practices (Kim et al., 2010). As such, improving our explicit understanding of the antecedents of early harsh parenting is crucial (Belsky & Jaffee, 2006; Hajal et al., 2015). Here, extending previous one-child-per-family research, we include two children per family in order to examine family-wide and child-specific predictors of harsh parenting at child age two years. Importantly, data are collected prospectively such that we account for age-related biases in differential treatment.

## Determinants of Parenting

Belsky’s (1984) influential theoretical framework for understanding predictors of parenting behavior and its recent update (Taraban & Shaw, 2018) converge on three broad categories of key parenting determinant: Child characteristics, parental personal resources, and contextual factors. A wealth of research supports these frameworks, including in clinical and community samples. In particular, child characteristics (e.g., gender, temperament), as well as maternal mental health, maternal level of education and partner support have been shown to have robust links with maternal parenting (Belsky & Jaffee, 2006; Taraban & Shaw, 2018). We include all of these predictor variables in our study of harsh maternal parenting in toddlerhood.

Including child characteristics in models for understanding parenting is important since it underlines the bidirectional nature of parenting, as first highlighted by Bell (1968). Empirical studies suggest that infants with “easier” temperaments, that is those who are sociable, adaptable, and easy-to-sooth, are more likely to experience responsive and warm parenting, whereas more “difficult” temperaments such as high impulsivity and low effortful control are consistently linked with parenting stress and parental harshness (Kiff et al., 2011; Oddi et al., 2013). In this context, child gender may also be important, as demonstrated using observational measures in a case-control sub-sample of the Avon Longitudinal Study of Parents and Children (ALSPAC), where boys at 12-months were shown to receive more negative and fewer positive parenting interactions than were girls (Thomson et al., 2014). Similarly, Ciciolla and colleagues (2013) observed that mothers of girls were more sensitive than mothers of boys in low-demand parenting tasks, while Bornstein and colleagues (2008) observed that Italian, Argentine and US mothers of toddlers were more sensitive and optimally-structuring with their daughters than with their sons.

In terms of maternal characteristics, psychopathology has been a key focus for parenting research, with consistent findings of direct and indirect effects on both parenting behaviors and adverse child outcomes (Cummings et al. 2005). Maternal depression and anxiety have been of central interest, and maternal depressive symptoms are well established as a risk factor for poorer parenting in the early years (Lovejoy et al., 2000). For example, maternal depression has been associated with higher rates of conflict during teaching tasks (Caughy et al., 2008), as well as being shown to interfere with maternal sensitivity and mother-infant attachment (Bernard et al., 2018), and to predict increased parenting stress (Bailhache et al., 2018). Similarly, maternal anxiety has been recognized as critical for parenting (Feldman, 2007; Kendler et al., 1997), predicting lower warmth and greater hostility (Seymour et al., 2014).

Another maternal characteristic that has received considerable attention is age. Research has suggested that younger motherhood may be associated with more negative parenting interactions (Scaramella et al., 2008; Thomson et al., 2014; Van Vugt et al., 2016). Importantly, this is considered specifically in connection with socioeconomic status (SES), a construct typically encompassing parental education, income and occupational factors, and considered a key contextual stressor for parenting. The relationship between young entry into parenthood and low SES is complex and bidirectional (Conger & Donnellan, 2007; Trenatacosta et al., 2010). Nevertheless, young mothers have been found more likely to come from lower SES backgrounds than their older counterparts, and to continue to be of lower SES throughout parenthood (Trenatacosta et al., 2010). Of interest is parental education, frequently used as a proxy for family SES in developmental research (Bornstein & Bradley 2012), as well as specifically and directly associated with negative discipline practices such as harshness and physical discipline (Bøe et al., 2014). As such, maternal age and maternal educational attainment are essential variables to consider here, both as markers of contextual socioeconomic stress and as maternal characteristics that may be predictive of harsh parenting independently.

Finally, the contextual factor of social support, commonly conceptualized as emotional, instrumental (e.g., financial support; Barnett et al. 2015) and informational support (e.g., advice, connection to services), has been found to be a key determinant of parenting, as both as a direct effect and as a buffer against other stressors (Taraban and Shaw 2018). Social support has many sources, including extended families (Simons et al., 1993), communities and co-parents or other romantic partners. The role of partners for maternal parenting is complex, and research findings have been mixed. For example, partner emotional support characterised by listening responsiveness and the use of endearment terms, has been shown to potentially disrupt generational cycles of harsh and abusive parenting (Conger et al., 2013). Furthermore, over and above other forms of social support, maternal positive perceptions of partner emotional support have been associated with reduced post-natal parenting stress, in turn directly associated with less harshness in parenting practices (Sampson et al., 2015). Similarly, positive and supportive co-parent relationships have been shown to have a positive influence on children’s adjustment (e.g., Cabrera et al., 2012). In contrast, aggressive partners have been shown to have adverse effects on parenting and child outcomes (Graham et al., 2012); and conflictive interparental relationships can “spillover” into parenting, conferring more negative parent-child interactions (Pedro et al., 2012). Moreover, in the context of maternal coercive parenting, maternal perceptions of a positive co-parent relationship have been shown to intensify the toxicity of this negative maternal parenting style for children’s behavioral outcomes (Latham et al., 2017).

## Family-wide vs Child-specific Factors

Behavioral-genetic studies suggest that, for the majority of child outcomes (including personality and psychopathology), two children growing up in the same family are hardly more similar than children selected at random from the population (Dunn & Plomin, 1990; Plomin, 2011). This implies that child-specific rather than family-wide influences are key for individual differences in outcomes – that is, within-family processes operate to differentiate siblings from one another rather than making them similar. For research, it follows that it is necessary to examine parenting behaviors towards more than one child in a family, enabling the identification of the comparative influences of family-wide determinants of parenting (e.g., parental mental health or educational attainment), in contrast to child-specific parenting determinants (e.g., child gender or temperament). Given that the parent-child relationship is reciprocal, it is unlikely that any parent treats two children in the same family in exactly the same way (Sameroff, 2010); assessing more than one child in the family facilitates the consideration of these likely differences to examine child-specific parenting, commonly referred to as “parental differential treatment”.

Addressing family-wide and child-specific family processes simultaneously is made statistically possible by multi-level modeling (MLM; see Jenkins et al., 2009). Although there are a number of papers that have considered MLM approaches to understanding the role of parental differential treatment for child outcomes, we are aware of only three papers that consider determinants of between- and within-family parenting per se within the MLM framework (Browne et al., 2012; Jenkins et al., 2003; O’Connor et al., 2006). These studies examined determinants of both positive (e.g., warmth, support) and negative (e.g., conflict, negative control) parenting using a range of methods including self report, interviews and observations. Child characteristics (e.g., age, gender, temperament), maternal and paternal characteristics (e.g., personality, emotional stability), and contextual factors (e.g., SES, inter-parental relationships, family-configuration) were found to influence the extent to which siblings within families were differentially treated by their parents. All three of these MLM studies examined differential parental treatment between siblings, and provided an important cross-sectional snapshot of the family when the children were at different chronological ages and developmental stages. Here, we seek to extend this literature using a prospective, longitudinal design, with child assessments collected as each child in the family in turn was two years old. In so doing, we are able to to consider the prediction of *direct* child-specific parenting – rather than the extent of differential treatment between siblings – reducing age-biased parental treatment effects (Dunn et al., 1986), using an MLM framework to study within- and between- family differences simultaneously. The novelty of the current study thus lies in its focus on predicting early harsh parenting of two children within a family, using a prospective, longitudinal, population-based design. We address three hypotheses:

1. Mothers would report substantial similarity in their use of harsh parenting between their two children, but child-specific harsh parenting would also be significant, and moderate in magnitude.
2. Boys would be the recipients of more harsh parenting than girls, as would children with more difficult temperaments in comparison to those with easier temperaments.
3. Harsh parenting would be more prevalent for younger mothers, those with more internalizing symptoms (i.e., depression, anxiety), those with lower levels of education, and mothers who perceived less support from their partners.

## Method

### Sample and Procedure

ALSPAC (http://www.bris.ac.uk/alspac) is an ongoing population-based study designed to investigate the effects of a wide range of factors on children’s health and development. All women resident in Avon, UK, who had expected dates of delivery between April 1, 1991 and December 31, 1992 were contacted and eligible for participation. The study cohort consisted of 14,541 pregnancies and 13,988 children alive at 12 months of age. The ALSPAC sample is described in detail elsewhere (Boyd et al., 2013; Copeland et al., 2009; Dunn et al., 2011).

Due to the recruitment window, a minority of ALSPAC families had more than one child who were part of the cohort of target children, and these families are the focus of the current study. We excluded identical twins because, unlike typical siblings, they share 100% of their segregating genes, but included fraternal twin pairs as they are only as similar genetically as are brothers and sisters. 234 eligible families formed the sampling frame for study, for whom data were available for 182 families, comprising 72 fraternal twin pairs and 110 sibling pairs (mean age gap = 15.50 months, range = 10–20 months). This sibling subsample did not differ significantly from the main ALSPAC sample with harsh parenting data available at 2 years in terms of child gender (ALSPAC, boys = 51.9%, sibling subsample, boys = 50.3%, χ^2^ = 0.36, *p* = 0.547), or family ethnicity (ALSPAC, self-identified as “white” = 97.9%, sibling subsample, “white” = 98.8%, χ^2^ = 3.70, *p* = 0.883). However, the sibling subsample was significantly younger than the main sample for maternal age (M_ALSPAC_ = 28.64, M_Sibling-subsample_ = 28.02, *t*(10418) = 2.48, *p* = 0.013), and level of educational attainment, with fewer mothers in the sibling subsample reaching O’level or above, educational achievement equivalent of a US High School Diploma or above (M_ALSPAC_ = 74.1%, M_Sibling-subsample_ = 66.8%, χ^2^ = 9.37, *p* = 0.002).

### Measures

#### Outcome

##### Harsh parenting

Harsh parenting at child age two-years was assessed by maternal report. Mothers were asked how often they slapped the child and how often they shouted at them. These two questions pertain to the dimensions of corporeal punishment/physical assault and psychological aggression on the Parent–Child Conflict Tactics Scales (Straus et al., 1998) and have been used as indicators of the harsh parenting construct in the ALSPAC sample and elsewhere (e.g., Meehan et al., 2017; Oliver et al., 2014). Responses on a five-point Likert scale were recorded from “never” (scored 0) to “every day” (scored 4), and items were summed (range 0–8; α = 0.67). Note that these reports were up to 20 months apart for siblings, depending on the age gap between the children.

#### Child-specific measures

##### Child temperament

Child temperament was measured in infancy (4 weeks), using 11 items assessing a range of positive and negative temperament characteristics. Mothers were asked to indicate how like the description their baby was on a five-point Likert scale, “very unlike” (0) to “very like” (4). Factor analysis indicated two factors classified here as “easy” and “difficult” temperament. “Easy” temperament included six items, “communicative”, “cuddly”, “active”, “sociable”, “happy” and “alert” (α = 0.73); “Difficult” temperament included five items, “grizzly”, “fretful”, “demanding”, “angry”, and “stubborn” (α = 0.76).

#### Family-wide measures

##### Maternal educational attainment

Maternal educational attainment was indexed by mothers’ self-reported highest educational qualification.

##### Maternal internalizing symptoms

Maternal internalizing symptoms were measured using The Crown-Crisp Experiential Index, used to measure psychopathology in community settings (Crown & Crisp, 1979), and includes subscales, each of eight items for anxiety (e.g., “Do you sometimes feel panicky?”) and depression (e.g., “Do you find yourself needing to cry?”). Respondents are asked to indicate how often they feel this way on a Likert scale from “I never feel this way” (1) to “This is exactly how I feel” (4). Data were collected at child age 8 weeks, 8 months and 21 months, and a sum score for each of maternal anxiety and depression calculated within time, before averaging across the three time points (Anxiety α = 0.83; Depression α = 0.82). Since maternal anxiety and depression correlated highly (*r* = 0.80), we created a mean score of maternal internalizing symptoms from these subscales.

##### Partner support

Partner support was measured using a total of nine items assessed at child ages 8 weeks, 8 months and 21 months, (e.g., “My partner provides the emotional support I need”, “I’m worried that my partner might leave me (reversed)”, and “If I feel tired I can rely on my partner to take over”). Items were rated by mothers on a four-point Likert scale from “I never feel this way” (0) to “This is exactly how I feel” (4). A within-time sum score was calculated, before averaging across the three time points (α = 0.82).

### Analyses

The multilevel modeling (MLM) framework allows us to account for our nested data structure (children nested within families), such that child- and family-level data can be analyzed simultaneously. MLM partitions between- and within-family variance and affords the inclusion of predictor variables that may contribute to the explanation of these variances. Using MLM with family data in this way yields fixed effects that are similar to traditional regression coefficients and random effects that represent estimates of child- and family-level variance (Jenkins et al., 2009).

Our first, baseline, model (Model 1) identified the degree of sibling similarity for harsh parenting. Specifically, the between-family component of Model 1 indicates the extent to which maternal harshness reported towards both siblings in a family is similar, but different from children other families. In contrast, the within-family component indicates the extent of parental differential (harsh) treatment towards children in the same family (see Jenkins et al., 2009). Model 2 included the child-specific level predictors of child gender and temperament. Model 3 included the family-wide predictors of maternal education, maternal internalizing symptoms, and perceived partner support. All child-specific and family-wide predictor variables were included in Model 4, in order to assess the overall variance explained, and the statistical independence of each predictor.

## Results

### Preliminary Analysis

Descriptive statistics and correlations among study measures are given in Table 1. There were no significant mean child gender differences (results available on request).

**Table 1:** Descriptive statistics and correlations for all measures

	Descriptives		Correlations									
	M	SD	1		2		3		4	5	6	7
*Outcome*			OS	YS	OS	YS	OS	YS				
1. Harsh Parenting												
Older Sibling	4.55	1.72	1	0.68***	−0.10	−0.05	0.05	−0.04	−0.12	−0.06	0.12	−0.21**
Younger Sibling	4.41	1.60		1	−0.11	−0.00	0.03	0.01	−0.18*	0.02	0.05	−0.10
*Within Family Level*												
2. Easy temperament												
Older Sibling	3.24	0.52			1	0.47***	−0.09	−0.19*	0.03	0.03	−0.23**	0.20**
Younger Sibling	3.08	0.64				1	−0.14	−0.22**	−0.08	−0.10	−0.09	0.10
3. Difficult temperament												
Older Sibling	1.26	0.90					1	0.30***	−0.02	−0.00	0.29***	−0.15
Younger Sibling	1.25	0.84						1	−0.01	−0.02	0.07	−0.26**
*Between Family Level*												
4. Maternal age (years)	27.22	4.41							1	0.23**	0.05	−0.01
5. Maternal educational attainment	3.02^a^	1.12								1	0.06	0.03
6. Maternal internalizing symptoms	−0.01	0.95									1	−0.44***
7. Partner support	20.09	5.11										1

Note: Maternal internalizing symptoms: mean of standardised anxiety and depression scores; Descriptives use unstandardized variables; correlations shown use standardized variables; ***p < 0.001; **p < 0.01; *p < 0.05

*OS* older sibling, *YS* younger sibling, *M* mean, *SD* standard deviation

^a^Equivalent of O’Levels

### Between- and Within-Family Variance in Harsh Parenting

The results from the MLM analyses are presented in Table 2. Model 1 yielded the intraclass correlation (ICC), calculated as the between-family variance divided by the total variance, representing sibling similarity in parental harshness. The ICC was 0.631/ (0.631 + 0.289) = 0.69, indicating that 69% of the variance in harsh parenting resided between families, and 31% within families. In other words, there was considerable sibling similarity in harsh parenting, as well as a significant, moderate amount of harsh parenting variance within families, confirming our first hypothesis.

**Table 2:** Fixed and random effects for models predicting maternal harsh parenting

	Parameter (SE)			
	Model 1	Model 2	Model 3	Model 4
Fixed effects				
*Within-Family*				
Child gender		0.02 (0.07)		0.04 (0.07)
Child easy temperament		0.01 (0.04)		0.04 (0.04)
Child difficult temperament		0.05 (0.04)		0.06 (0.05)
*Between-Family*				
Maternal age			−0.17* (0.07)	−0.17* (0.08)
Maternal educational attainment			0.01 (0.07)	−0.00 (0.07)
Maternal internalizing symptoms			0.018 (0.08)	−0.01 (0.08)
Partner support			−0.16* (0.08)	−0.21* (0.08)
Random effects				
Within-family	0.29***	0.26***	0.29***	0.26***
Between-family	0.63***	0.63***	0.56***	0.58***
RMSEA	0.00	0.00	0.00	0.00
AIC	833.42	742.05	748.91	683.38
*χ*^2^	0.000 (0) *p* < 0.001	1.308 (3) *p* = 0.727	10.900 (4) *p* = 0.028	14.590 (7) *p* = 0.042

Note. **p < 0.001; *p < 0.01; *p < 0.05; Model 1 = baseline

### Prediction of Harsh Parenting

Contrary to our expectations, but congruent with the preliminary correlations, none of the child-specific factors significantly predicted harsh parenting (Model 2). In line with our hypothesis, both maternal age and perceived partner support provided significant independent prediction of harsh parenting (Model 3). Specifically, older mothers reported less harsh parenting (effect size = 0.177), as did those with partners perceived as more supportive (effect size = 0.206). However, contrary to our hypothesis, maternal internalizing symptoms did not significantly predict harsh parenting (Model 3). In combination, family-wide predictors accounted for 11% (1-(0.563/0.631)) of the between-family variance in harsh parenting. These results were confirmed by the final model, which included both the child-specific and family-wide predictors (Model 4).

## Discussion

To our knowledge, the current study is the first longitudinal study to test family-wide and child-specific predictors of harsh parenting simultaneously in early toddlerhood, using a prospective design to reduce age-related parental differential treatment. Using a population-based sampling frame in the UK, we found that younger maternal age and maternal perceptions of less partner support predicted maternal harsh parenting over and above child gender and temperament, maternal internalizing symptoms and maternal educational attainment.

Previous MLM studies describing parenting determinants (Browne et al., 2012; Jenkins et al., 2003; O’Connor et al., 2006) have examined differential parenting of siblings within the same family. However, the prospective nature of ALSPAC data allows us to add to this literature by examining family processes as each child in turn is two-years old rather than a cross-sectional snapshot of the family when the children are at different chronological ages and developmental stages. In this way, a major study-strength is that we were able to reduce the confound of age-biased parental differential treatment (Dunn et al., 1986). For example, MLM studies using a wider range of child ages have shown that, while there is often substantial similarity in the way that siblings are treated by parents (Jenkins et al. 2003; O’Connor et al., 2006), each year of sibling age difference confers a drop in negative parenting measures (Jenkins et al. 2003), perhaps due to older children’s increased autonomy and reduced need for discipline. Thus, having reduced this developmental confound in the current study, our expectation was that children within the same family would receive markedly similar levels of harsh parenting, and this was substantiated. However, given our use of mothers’ reports of their own harsh parenting with siblings no more than 20 months apart, the extent of variance in harsh parenting that also resided within families was noteworthy.

Counter to expectation was our finding that child gender did not explain a significant amount of the variance in harsh parenting. However, prior MLM findings of child gender associations with differential parenting are mixed (Jenkins et al., 2003; O’Connor et al., 2006), and our results are in line with other studies that report no gender effects in harsh parenting at this young child age (Kim et al., 2010). Also unexpected was the lack of prediction by child temperament: maternal perceptions of infant “difficulty” or lack of infant “ease” in the current study, did not place children at heightened risk for harsh parenting. Given the very early (4-weeks old) temperament measure used here, we posit that this may be due to the relative instability of child temperament in the first months of life (Beekman et al., 2015). Indeed, child temperament is thought to stabilize later in development (Roberts & Del Vecchio, 2000) and may be a stronger predictor of later child outcomes and parenting behaviors from 9−18 months (Planalp & Goldsmith, 2019). Moreover, temperamental factors may play a greater role in parenting at older ages with increased child autonomy and non-compliance (Kuczynski & Kochanska, 1990), which may explain findings in the previous MLM differential treatment literature (Jenkins et al., 2003; O’Connor et al., 2006).

Turning to the larger portion of variance, that is, between-family variance in harsh parenting, our prediction of harsh parenting from maternal age, with younger age predicting higher levels of harsh parenting over and above maternal education, was striking. Importantly, although the sample of mothers included were on average younger than the population sample from which they were drawn, this sub-sample did not comprise mothers who would all be considered of young maternal age. Nevertheless, we posit that our maternal age variable was a marker of the sample’s social circumstances – mothers with two children born within a short time frame – that may be important for parenting. Indeed, rapid repeat births (siblings born within 24 months) are more common among younger mothers and mothers from lower socioeconomic groups (Norton et al., 2017), and have been shown to predict neglectful parenting practices and poor child outcomes (Crowne et al., 2012). Furthermore, the maternal personality and behavioral characteristics that predict earlier entry into parenthood (Woodward et al., 2006) and rapid repeat births (Crittenden et al., 2009) may also have direct (Trentacosta et al., 2010) and indirect influence on harsh parenting (Moffitt et al., 2002).

In line with theoretical models (Belsky, 1984; Taraban & Shaw, 2018), maternal perceptions of partner support was the other elucidated predictor of maternal harsh parenting in the current study, with maternal perceptions of higher levels of support predicting lower levels of harsh parenting. These findings fit well with some of the existing research demonstrating the influence of inter-parental relationships on parenting (Conger et al., 2013; Pedro et al., 2012; Sampson et al., 2015), not least within an MLM framework, where higher levels of parental negativity have been found in families with lower socioeconomic status and higher marital dissatisfaction (Jenkins et al., 2003). Relatedly, between-family variation in mother-child conflict has been shown to be explained by family-stress factors including single-parenthood (O’Connor et al., 2006). We speculate that an increased focus on the inter-partner relationship due to its unsupportive and potentially conflictual nature, may have limited the personal resources of our mothers, heightening parenting stress, and thus contributing to the use of harsher parenting practices (Conger et al., 2013; Jenkins et al., 2003).

Another point of note in our study is that maternal internalizing symptoms did not predict harsh parenting, contrary to our hypothesis and previous research (e.g., Bernard et al., 2018; Caughy et al., 2008; Lovejoy et al., 2000; Seymour et al., 2014), including in the MLM context (O’Connor et al., 2006). One possible reason for our findings may be due to our sample of community mothers. A recent meta analysis noted that the overall effect size for associations between maternal depression and parenting behaviors was small, with single community samples of mothers showing smaller effect sizes than clinical samples (Bernard et al., 2018). The authors posited that clinical levels of depression may be more strongly associated with negative parenting than non-clinical levels. Similarly, Seymour and colleagues (2014) note that their sample showed significantly more anxiety symptoms than a normative population sample, with 18% of their mothers reporting mild to extremely severe levels of anxiety, many with comorbid depression symptoms. This could mean that internalizing symptoms among our sample were not severe enough to predict harsh parenting behavior and that it is clinical levels of depression and anxiety that place parents at higher risk for harsh parenting practices. It is possible that levels of low mood and anxiety found in non-clinical samples are associated with less parental positivity, but that they are not predictive of the harsh parenting approaches considered most deleterious for child outcomes is likely positive news for children’s wellbeing.

While internalizing problems did not predict parental harshness in our study, it was interesting that maternal internalizing symptoms were moderately and negatively correlated with maternal perceptions of partner support. This could reflect positive affect associations with more positive relationships, as well as previous research finding that women with depression may be less able to obtain a high level of social support and to avoid social conflicts (Finch & Graziano, 2001). While causality cannot be established, it may be that partners of mothers with higher levels of internalizing symptoms feel less able to support them, or perceptions of lower levels of support contribute to maternal internalizing symptoms, particularly in the post-partum period (see Dennis & Ross, 2006). Exploring these issues further may be an important avenue for future research.

### Limitations and Future Directions

The findings of our study must be viewed in light of study limitations. In particular, as a secondary analysis, we were limited to the relevant data available. For example, in considering just two children in each family, and two children born close together, we potentially over-simplify family sub-systems and processes that may be important for parenting practices and ultimately for child adjustment. Including families of three or more children in studies like ours may uncover more complex processes lying at the heart of differential treatment, such that the generalizability of our findings may be limited (O’Connor et al., 2006). We also acknowledge that our measure of harsh parenting was limited with just two relevant questions available at this age. Despite the face validity of the items, further research using more detailed and rigorous measures is warranted to afford more nuanced understanding of the determinants harsh parenting experiences between siblings. Relatedly, our exclusive use of mother-report is limited and it is possible that child-specific factors would be better identified using independent reports since our mother-reported family-wide factors (most notably partner support and maternal internalizing symptoms) were associated with their reports of child characteristics. Observations and self-reports of parenting show small to moderate associations with each other (Hendricks et al., 2018), and offer distinct information for understanding diverse child adjustment outcomes (Schofield et al., 2016). As such, a full picture of these family processes is best gained from multiple sources of information (Dunn & Kendrick, 1980), which were not available in our data. Importantly, however, for harsh parenting, any social desirability biases would likely mean under-reporting of these parenting practices by mothers, and – pertinent to the current prospective study – self reports have the advantage of capturing parenting practices over time, rather than observations that are based on short and intermittent visits under specific conditions (Waylen et al., 2008). Further, for reasons of power, the current study was constrained to mothers, despite the fact that paternal harsh parenting is a key family factor, and the determinants of paternal parenting are crucial to understand. Future research would be well-placed to consider paternal parenting, which may be differentially predicted by within- and between-family factors (Hajal et al., 2015). Finally, we were unable to elucidate whether the parenting differences we capture are differences in mothers’ general use of harsh parenting over time. It is plausible that harsh parenting may increase or decrease as a function of having a second child and/or more experience with the parenting role; relatedly, for reasons of power, we do not distinguish between families with fraternal twins and families with siblings, which may have masked more nuanced effects. These are important questions for future research.

### Implications

Within our conservative framework, the importance of examining child-specific and family-wide parenting determinants simultaneously is highlighted. Here, we find the roles of maternal age and perceptions of partner support for harsh parenting practices to dominate, over and above child-specific factors such as gender and early temperament, and maternal internalizing symptoms. Our findings suggest that targets for positive parenting support may usefully be younger mothers as well as those with young children close in age. Importantly, our findings also point to the importance of improving mothers’ support – or perceptions of support – from partners. In particular, the results endorse the increasing emphasis on father involvement in the perinatal period which may be an excellent platform to deliver education on the integral role of partner support in the transition to parenthood (May & Fletcher, 2013; Parry et al., 2019). Further, this study supports the heightened interest in supporting the inter-parental relationship in order to improve positive outcomes for children (e.g., Department for Work and Pensions 2019). Our results bolster the need for more research that considers both between and within-family parenting variances to better understand determinants of harsh parenting and to further inform approaches to supporting maternal wellbeing and offereing family support.
